# Associations of Sedentary Behaviour, Physical Activity, Blood Pressure and Anthropometric Measures with Cardiorespiratory Fitness in Children with Cerebral Palsy

**DOI:** 10.1371/journal.pone.0123267

**Published:** 2015-04-02

**Authors:** Jennifer M. Ryan, Owen Hensey, Brenda McLoughlin, Alan Lyons, John Gormley

**Affiliations:** 1 School of Medicine, Trinity College Dublin, Ireland; 2 Institute of Environment, Health and Societies, Brunel University London, United Kingdom; 3 Central Remedial Clinic, Dublin, Ireland; 4 Department of Physiotherapy, Enable Ireland, Cork, Ireland; School of Public Health, Zhejiang University, CHINA

## Abstract

**Background:**

Children with cerebral palsy (CP) have poor cardiorespiratory fitness in comparison to their peers with typical development, which may be due to low levels of physical activity. Poor cardiorespiratory fitness may contribute to increased cardiometabolic risk.

**Purpose:**

The aim of this study was to determine the association between sedentary behaviour, physical activity and cardiorespiratory fitness in children with CP. An objective was to determine the association between cardiorespiratory fitness, anthropometric measures and blood pressure in children with CP.

**Methods:**

This study included 55 ambulatory children with CP [mean (SD) age 11.3 (0.2) yr, range 6-17 yr; Gross Motor Function Classification System (GMFCS) levels I and II]. Anthropometric measures (BMI, waist circumference and waist-height ratio) and blood pressure were taken. Cardiorespiratory fitness was measured using a 10 m shuttle run test. Children were classified as low, middle and high fitness according to level achieved on the test using reference curves. Physical activity was measured by accelerometry over 7 days. In addition to total activity, time in sedentary behaviour and light, moderate, vigorous, and sustained moderate-to-vigorous activity (≥10 min bouts) were calculated.

**Results:**

Multiple regression analyses revealed that vigorous activity (β = 0.339, p<0.01), sustained moderate-to-vigorous activity (β = 0.250, p<0.05) and total activity (β = 0.238, p<0.05) were associated with level achieved on the shuttle run test after adjustment for age, sex and GMFCS level. Children with high fitness spent more time in vigorous activity than children with middle fitness (p<0.05). Shuttle run test level was negatively associated with BMI (r^2^ = -0.451, p<0.01), waist circumference (r^2^ = -0.560, p<0.001), waist-height ratio (r^2^ = -0.560, p<0.001) and systolic blood pressure (r^2^ = -0.306, p<0.05) after adjustment for age, sex and GMFCS level.

**Conclusions:**

Participation in physical activity, particularly at a vigorous intensity, is associated with high cardiorespiratory fitness in children with CP. Low cardiorespiratory fitness is associated with increased cardiometabolic risk.

## Introduction

Cerebral palsy (CP) is a neurodevelopmental condition that begins in childhood but has a life-long impact on the individual. Children with CP have reduced levels of everyday physical activity and poor cardiorespiratory fitness in comparison to their peers with typical development [1–4]. In 2003, the American Physical Therapy Association’s Section on Pediatrics and its Research committee highlighted the critical need to identify and promote effective interventions to improve cardiorespiratory fitness in children with CP [5]. In combination with the increased cost of locomotion due to motor impairments and orthopedic conditions, reduced cardiorespiratory fitness increases the physical strain associated with walking in children with CP [6]. Adults with CP cite poor endurance as a reason for deterioration in walking function and premature loss of mobility [7]. In addition, low cardiorespiratory fitness is associated with obesity, hypertension, dyslipidemia, insulin resistance and the metabolic syndrome in children with typical development [8–10]. It is unknown, however, if a similar relationship exists in children with CP.

The negative consequences of poor cardiorespiratory fitness indicate that children with low fitness should be identified and methods to increase fitness should be implemented. Previous studies have shown that exercise interventions result in short-term improvements in cardiorespiratory fitness among children with CP [11,12]. However there is limited evidence to suggest that exercise interventions result in long-term health behavior change and maintenance of adequate cardiorespiratory fitness. Information is required about the association between habitual physical activity, sedentary behaviour and cardiorespiratory fitness in children with CP in order to optimise physical activity recommendations to increase and maintain fitness in children with CP.

In the general population increased time in physical activity and reduced time in sedentary behaviour is associated with cardiorespiratory fitness [13]. Only three studies have investigated the relationship between physical activity and cardiorespiratory fitness in children and adults with CP, respectively [14–16]. All three studies failed to find an association. This may be because total physical activity or total walking activity, rather than physical activity intensity was measured in these studies. In children with typical development, time spent in certain components of physical activity intensity, particularly vigorous physical activity, is strongly associated with cardiorespiratory fitness [17–19]. The relationship between sedentary behaviour, physical activity intensity and cardiorespiratory fitness has not been investigated in children with CP. The aim of this study was to determine the association between sedentary behaviour, physical activity intensity and cardiorespiratory fitness in children with CP. An objective was to investigate the association between cardiorespiratory fitness, anthropometric measures and blood pressure in children with CP. We hypothesised that increased time in moderate and vigorous physical activity and reduced time in sedentary behaviour would be associated with high cardiorespiratory fitness. We also hypothesised that low cardiorespiratory fitness would be associated with high body mass index (BMI), indicators of central adiposity and high blood pressure.

## Methods and Materials

### Participants

Seventy-one ambulatory children with CP age 6 to 17 years, classified in level I and II of the Gross Motor Function Classification System (GMFCS) [20] participated in this study. The GMFCS distinguishes between levels of motor function based on functional mobility and the need for assistive technology, particularly mobility aids. Children in GMFCS level I are able to walk indoors and outdoors without assistance and can perform gross motor skills such as running and jumping but speed, balance and coordination are reduced. Children in level II can also walk indoors and outdoors without assistance but have only minimal ability to perform gross motor skills like running and jumping. Participants were recruited from seven centers of varying geography within Ireland that provide services to children with disabilities as part of a larger study into the relationship between blood pressure, adiposity and physical activity in children with CP [21]. Exclusion criteria for the study were a severe intellectual disability or having undergone surgery in the previous six months. Ethical approval for this study was granted by the University of Dublin’s Faculty of Health Sciences’ ethics committee, the Central Remedial Clinic’s ethics committee and the Enable Ireland Research Ethics and Quality Committee. Written informed consent was obtained from participants’ guardians and written assent was obtained from children. Children who were unable to provide written assent provided verbal assent.

Participants attended their local physiotherapy department for testing. All measurements were taken on one occasion by a single researcher (JMR).

### Anthropometric Measures

Height (to the nearest 0.1 cm) was measured in bare feet at the end of a gentle inspiration using a portable stadiometer (Invicta Plastics Ltd., Leicester, England). Children were encouraged to straighten their hips and knees and lower their heels to the ground. They received assistance if required. Mass (to the nearest 0.1 kg) was measured in bare feet and light clothing using an electronic platform scale (SECA 635, Hamburg, Germany). Waist circumference (WC) was measured two times, to the nearest 0.1 cm, using the method recommended by the World Health Organisation [22], i.e. on bare skin midway between the lower rib margin and the iliac crest at the end of a gentle expiration. The mean of the two measurements was used in data analysis. Waist-height ratio (WHtR) was calculated as WC divided by height. BMI was calculated and converted to International Obesity Task Force (IOTF) grades (BMI grade) [23] using the “LMS Growth” Microsoft Excel add-in software [24].

### Blood Pressure

Blood pressure was measured from the right arm or the least affected side in the case of significant asymmetry, using the Omron 705 IT BP monitor. The Omron 705 IT has been validated as an accurate device for blood pressure measurement in children and adolescents [25]. The appropriate size cuff was chosen based on mid-arm circumference and placed so that the lower edge was 3 cm above the elbow crease and the bladder was centred over the brachial artery. Participants rested in a seated position with their back supported, legs uncrossed and arm supported, for at least five minutes before three measurements were obtained at a 1–2 min interval. The average of the last two measurements was used in data analysis. Systolic blood pressure (SBP) and diastolic blood pressure (DBP) were expressed as standard deviation scores (zSBP and zDBP, respectively) [26] to control for the variation in SBP and DBP between participants as a result of age, sex and height. Although the standard deviation scores used for SBP and DBP have not been validated in the CP population, in the absence of standard deviation scores for children with CP the use of standard deviation scores for children with typical development was considered the most reasonable approach.

### Cardiorespiratory Fitness

Cardiorespiratory fitness was measured using a 10 m shuttle run test. This test demonstrated excellent validity and reliability in children with CP [27]. Children were asked to walk between the two markers (10 m apart) at initial speeds of 5 km.h^-1^ for children classified in GMFCS level I and 2 km.h^-1^ for children in GMFCS level II. The speed increased by 0.25 km.h^-1^ every minute. The walking/running pace was determined by a series of beeps emitted from a CD. The children were assisted during the initial stages of the test to coordinate their running speed with the pace of the audio signal. If a child continued having difficulty pacing themselves they were accompanied throughout the test. The test ended when the child was more than 1.5 m away from the marker on two consecutive paced signals or the child refused to continue. Heart rate was measured using a Polar heart rate monitor. The criteria for a maximal test were a maximal heart-rate ≥180 bpm plus a subjective symptom such as shortness of breath [27]. In accordance with the standard protocol the test result was measured in units of a level (e.g. 12) or a half level (e.g. 12.5). Each level corresponds to one minute. In order to characterise each child’s cardiorespiratory fitness relative to children with CP of the same height, sex and GMFCS level, children were stratified into tertiles using reference centile curves for the 10 m shuttle run test developed on children with CP from the Netherlands, Switzerland, Australia, Canada and the United States [28].

### Physical Activity

Physical activity was measured with the RT3 accelerometer (Stayhealthy, Inc., Monrovia, CA, USA). The RT3 is a triaxial accelerometer, measuring accelerations in three planes. A resulting vector magnitude is calculated as the square root of the sum of squared activity counts for each plane. The RT3 provides count data for vector magnitude in 1-min epochs (counts.min^-1^). All participants were asked to wear the RT3 for 7 days on their right hip (or least affected side in the case of significant asymmetry) in the midaxillary line. Participants were told to wear the RT3 for waking hours and only to remove it for bathing and swimming. Participants were asked to record the times that they removed the monitor and the activities they completed while not wearing the monitor.

Data was downloaded from the RT3 using the RT3 Assist software (StayHealthy, Inc.) before being exported to and processed in Microsoft Excel 2010. Valid activity data was defined as having at least four days of at least 10 hours wear time per day. Sedentary activity was defined as <41 counts.min^-1^, light activity (LPA) was defined as 41–950 counts.min^-1^, moderate activity (MPA) was defined as 950–3410 counts.min^-1^, vigorous activity (VPA) was defined as >3410 counts.min^-1^, in accordance with the Vanhelst cut-points which have been validated in children with CP [29,30]. Data are presented as time in LPA, MPA, and VPA accumulated in 1 min bouts, and time in moderate-to-vigorous activity (MVPA) accumulated in 10 min bouts. When calculating sustained MVPA one minute below the moderate threshold was allowed for within a bout of ≥ 10 minutes of MVPA without the bout being deemed ended. However, if two or more minutes below the moderate threshold were recorded the bout was considered ended. Percentage time spent in sedentary activity (i.e. minutes spent in sedentary activity/total wear time) and mean activity counts.min^-1^ (an indication of total activity) are also presented.

### Statistical analysis

The Kolmogorov-Smirnov test was used to investigate the distribution of the data. Where possible, non-normally distributed variables were transformed using a square-root transformation to a normal distribution. Mean and standard deviations (SD) are presented for normally distributed continuous data. Median and interquartile ranges (IQR) are presented for non-normally distributed continuous variables.

Multiple linear regression analyses using the enter method were performed to investigate the independent associations between sedentary activity, physical activity and cardiorespiratory fitness. Specifically, in each model, the level achieved in the shuttle run test was entered as the dependent variable; percentage sedentary time, time spent in LPA, MPA, VPA and MVPA, and mean counts.min^-1^ were entered as separate potential predictors. Age, sex, and GMFCS level were all entered as standard covariates. Sex and GMFCS level were entered as indicator variables where 0 = female, 1 = male and 0 = GMFCS level I, 1 = GMFCS level II. In preliminary analyses, height and bilateral/unilateral involvement were evaluated as potential covariates but were not included in the final models; unilateral/bilateral involvement was not associated with shuttle run test level and height was not associated with shuttle run test level when age was included in the model.

These analyses were conducted a second time adjusting for mean counts.min^-1^ in addition to standard covariates in order to examine the extent to which total physical activity attenuated the relationship between physical activity intensity and cardiorespiratory fitness. Untransformed variables were used in all analyses as the distribution of the standardised residuals of variables was normal. Collinearity was assessed using the variance inflation factor (VIF). VIFs of ≤10 were considered acceptable [31].

Partial correlation coefficients were calculated to investigate the association between shuttle run test level and zSBP, zDBP, BMI grade, WC and WHtR, respectively, after adjustment for age, sex and GMFCS level. Further analyses investigated the relationship between shuttle run test level and blood pressure after adjustment for individual anthropometric measures, and the relationship between shuttle run test level and measures of central adiposity after adjustment for BMI grade.

Finally, a one-way analysis of variance was used to compare percentage sedentary time, LPA, MPA, VPA, MVPA, mean counts.min^-1^, BMI grade, WC, WHtR, zSBP, and zDBP across tertiles of cardiorespiratory fitness. Fisher’s least significant difference post-hoc tests were conducted as appropriate. BMI grade was not normally distributed across fitness tertiles and was therefore square-root transformed for this analysis. All statistical analyses were performed using SPSS, version 20, with α = 0.05.

## Results

Of the 71 children who completed the shuttle run test, four children did not meet the criteria for a maximal test and physical activity data was not obtained on twelve children; these sixteen children were removed from the analysis. Of the twelve children who did not have physical activity data, four children did not meet the criteria for valid wear-time and eight children returned monitors without any data because of interference with the monitor or battery malfunction. The final sample size was 55. Descriptive statistics are presented in Table 1. The mean level achieved on the shuttle run test was 9.0 (4.0) min. According to reference centile curves 8 children (14.5%), 24 children (43.6%), and 23 children (41.8%) were categorized as low, middle and high fitness, respectively. Children wore the accelerometer for a median of 7 (1) days and a mean time of 760.5 (55.2) min per day. Percentage time spent in sedentary activity, time spent in each component of physical activity, and mean counts.min^-1^, for the total sample and across fitness tertiles are presented in Table 2.

**Table 1:** Characteristics of participants (n = 55).

Males:females	34:21
Age, yr, mean (SD)	11.3 (0.2)
Height, cm, median (IQR)	140.5 (27.9)
BMI, kg.m^-2^, mean (SD)	18.4 (3.4)
BMI grade, mean (SD)	0.25 (1.16)
Waist circumference, cm, median (IQR)	65.0 (15.0)
Waist-height ratio, median (IQR)	0.43 (0.07)
Systolic blood pressure, mmHg, mean (SD)	109.5 (14.0)
zSBP, mean (SD)	-0.37 (1.21)
Diastolic blood pressure, mmHg, mean (SD)	64.1 (11.7)
zDBP, mean (SD)	0.84 (1.33)
GMFCS level, n (%)	
Level I	46 (83.6)
Level II	9 (16.4)
Classification of cerebral palsy, n (%)	
Spastic unilateral	38 (69.1)
Spastic bilateral	15 (27.3)
Non-spastic	2 (3.6)

zSBP, z-scores for systolic blood pressure; zDBP, z-scores for diastolic blood pressure; GMFCS, Gross Motor Function Classification System

**Table 2:** Percentage time spent in activity behaviour, time spent in each physical activity intensity, and mean counts.min^-1^ across cardiorespiratory fitness tertiles. Data presented as mean (SD).

Cardiorespiratory fitness tertile	Percentage sedentary time (%)	LPA (min)	MPA (min)	VPA (min)	MVPA (min)	Mean counts.min^-1^
Low	29.0 (7.7)	420.2 (33.9)	88.3 (4.3)	4.2 (1.0)	24.2 (4.4)	419.0 (152.3)
Middle	33.6 (12.8)	407.2 (65.3)	83.7 (5.7)	4.9 (2.2)*	28.0 (6.7)	399.2 (157.7)
High	32.8 (12.2)	406.9 (74.0)	95.3 (6.2)	10.6 (3.5)*	44.9 (9.7)	473.51 (203.9)
All	32.6 (11.9)	409.0 (65.0)	89.1 (5.6)	6.9 (2.8)	34.0 (8.0)	433.2 (178.2)

*Significant difference in physical activity intensity between tertiles, p<0.05

LPA, light physical activity; MPA, moderate physical activity; VPA, vigorous physical activity; MVPA, moderate-to-vigorous physical activity in 10 min bouts

Regression models to show how physical activity was associated with shuttle run test level are presented in Table 3. Total activity (mean counts.min^-1^), MVPA and VPA were positively associated with shuttle run test level. Percentage time spent in sedentary activity, LPA and MPA were not associated with shuttle run test level. Only VPA remained associated with shuttle run test level after adjustment for total activity.

**Table 3:** Multiple linear regression analyses to examine the contribution of vigorous physical activity, moderate-to-vigorous activity (10 min bouts) and total activity to level achieved on the shuttle run test.

Dependent Variable	R^2^	Independent variables	Standardised beta	p-value	VIF	F
Shuttle run test level (min)	0.595	Age	0.605	0.000	1.037	18.343*
		Sex	0.092	0.334	1.094	
		GMFCS level	0.326	0.001	1.037	
		Vigorous physical activity	0.339	0.001	1.048	
Shuttle run test level (min)	0.543	Age	0.610	0.000	1.038	14.823*
		Sex	0.102	0.317	1.104	
		GMFCS level	0.336	0.001	1.061	
		Moderate-to-vigorous activity (10 min bouts)	0250	0.015	1.087	
Shuttle run test level (min)	0.534	Age	0.679	0.000	1.150	14.298*
		Sex	0.122	0.228	1.079	
		GMFCS level	0.341	0.001	1.076	
		Mean counts.min^-1^	0.238	0.027	1.168	
Shuttle run test level (min)	0.623	Age	0.478	0.000	1.599	16.215*
		Sex	0.080	0.389	1.099	
		GMFCS level	0.277	0.004	1.121	
		Mean counts.min^-1^	-0.408	0.060	5.816	
		Vigorous physical activity	0.684	0.001	5.218	

*p<0.001; VIF, variance inflation factor; GMFCS, Gross Motor Function Classification System.

Shuttle run test level was negatively associated with BMI grade, WC, WHtR, and zSBP (Table 4). WC and WHtR respectively, remained associated with shuttle run test level after adjustment for BMI (Table 4). Shuttle run test level was not associated with zDBP. Shuttle run test level was not associated with zSBP after adjustment for WC, WHtR, or BMI grade.

**Table 4:** Relationship between cardiorespiratory fitness, systolic blood pressure, body mass index, waist circumference and waist-height ratio.

	Shuttle run test level	
	Partial correlation coefficient	p-value
zSBP^a^	-0.306	0.027
BMI grade^a^	-0.451	0.001
Waist circumference^a^	-0.560	0.000
Waist circumference^b^	-0.397	0.004
Waist-height ratio^a^	-0.560	0.000
Waist-height ratio^b^	-0.374	0.007

^a^adjusted for age, sex and GMFCS level

^b^adjusted for age, sex, GMFCS level and BMI grade; zSBP, z-scores for systolic blood pressure

**Fig 1:**
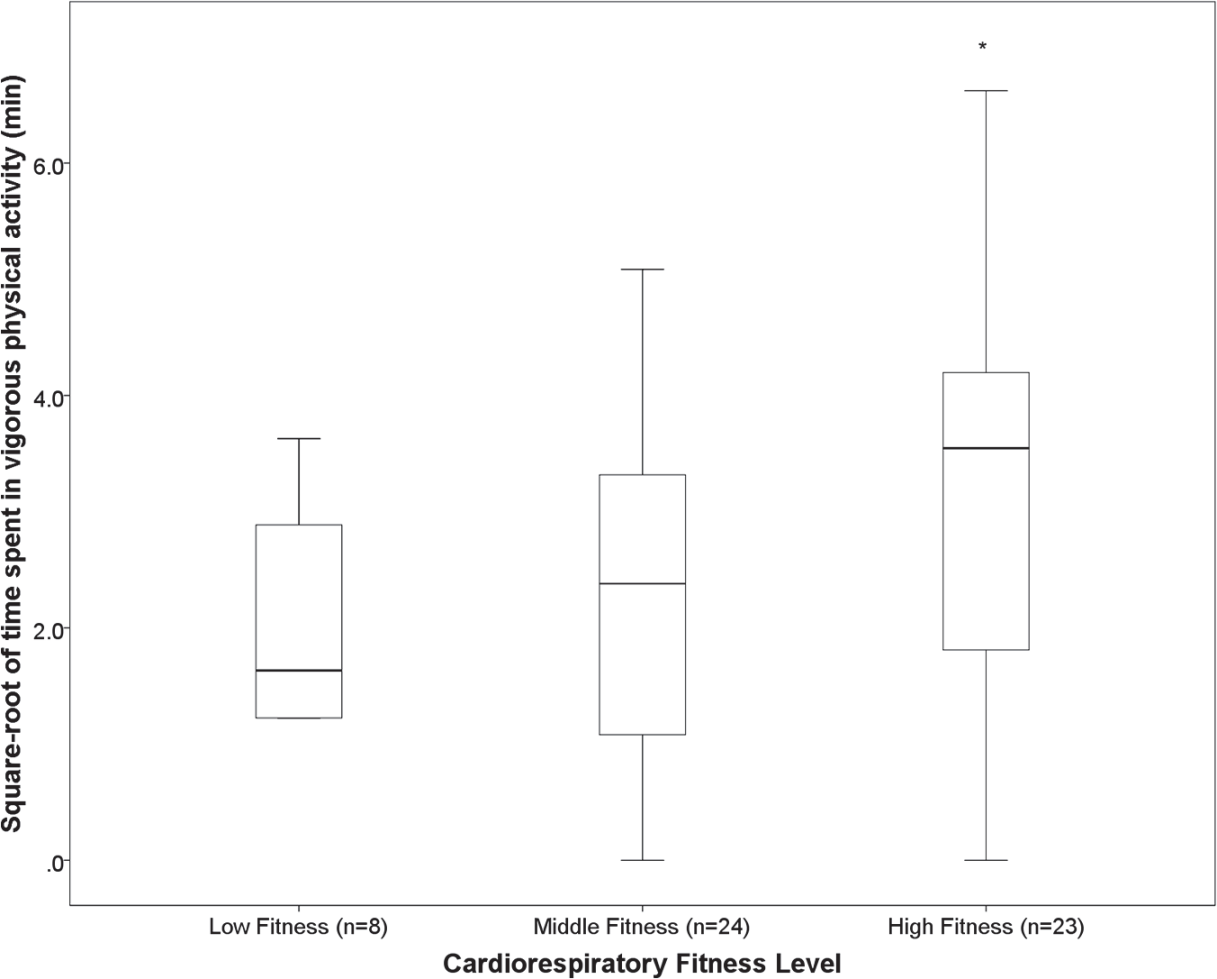
Mean vigorous physical activity across tertiles of cardiorespiratory fitness. *Significantly different to “Middle” fitness group, p<0.05.

**Fig 2:**
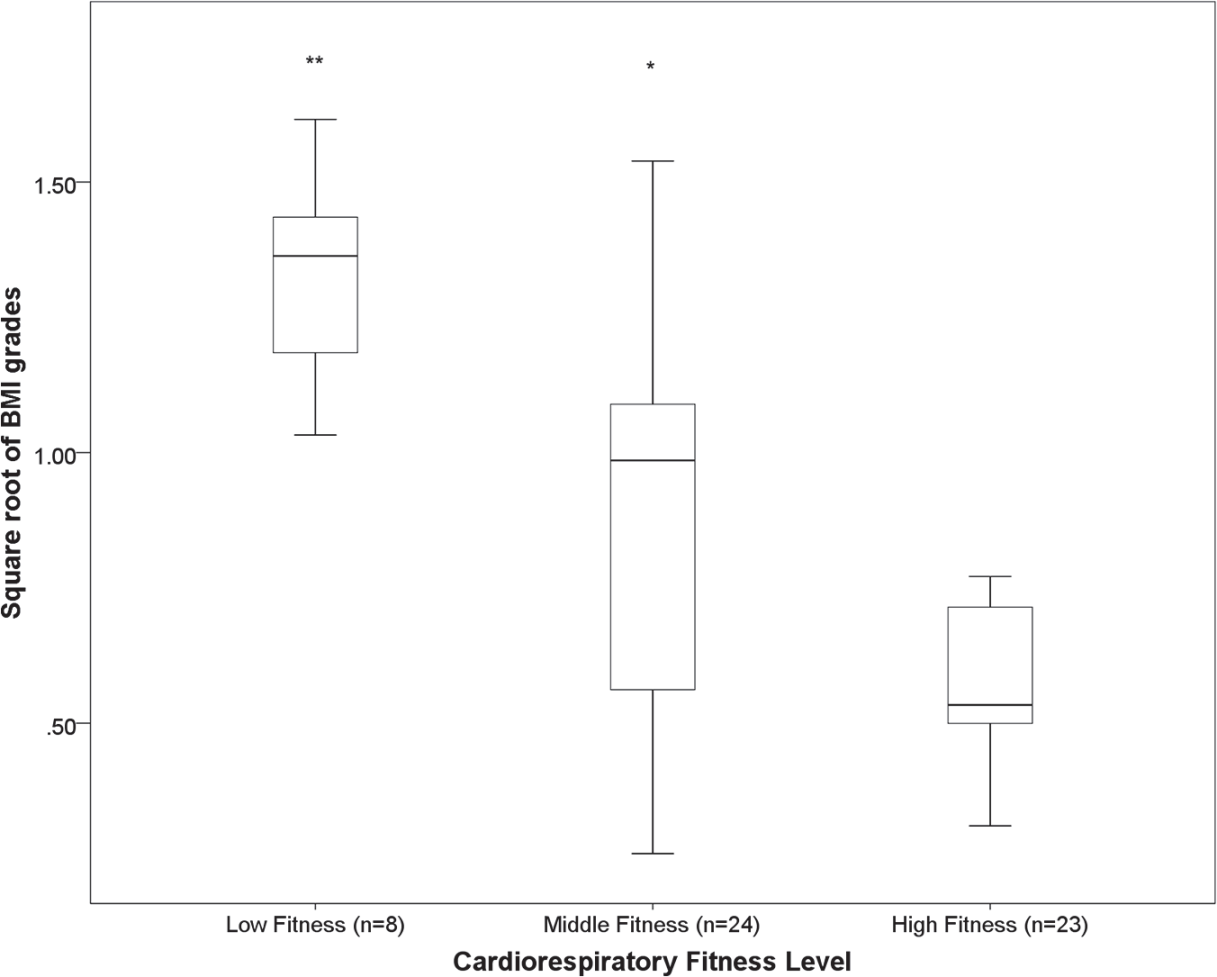
Mean BMI grade across tertiles of cardiorespiratory fitness. **Significantly different to “Middle” and “High” fitness group, p<0.01. *Significantly different to “”High” fitness groups, p<0.05.

**Fig 3:**
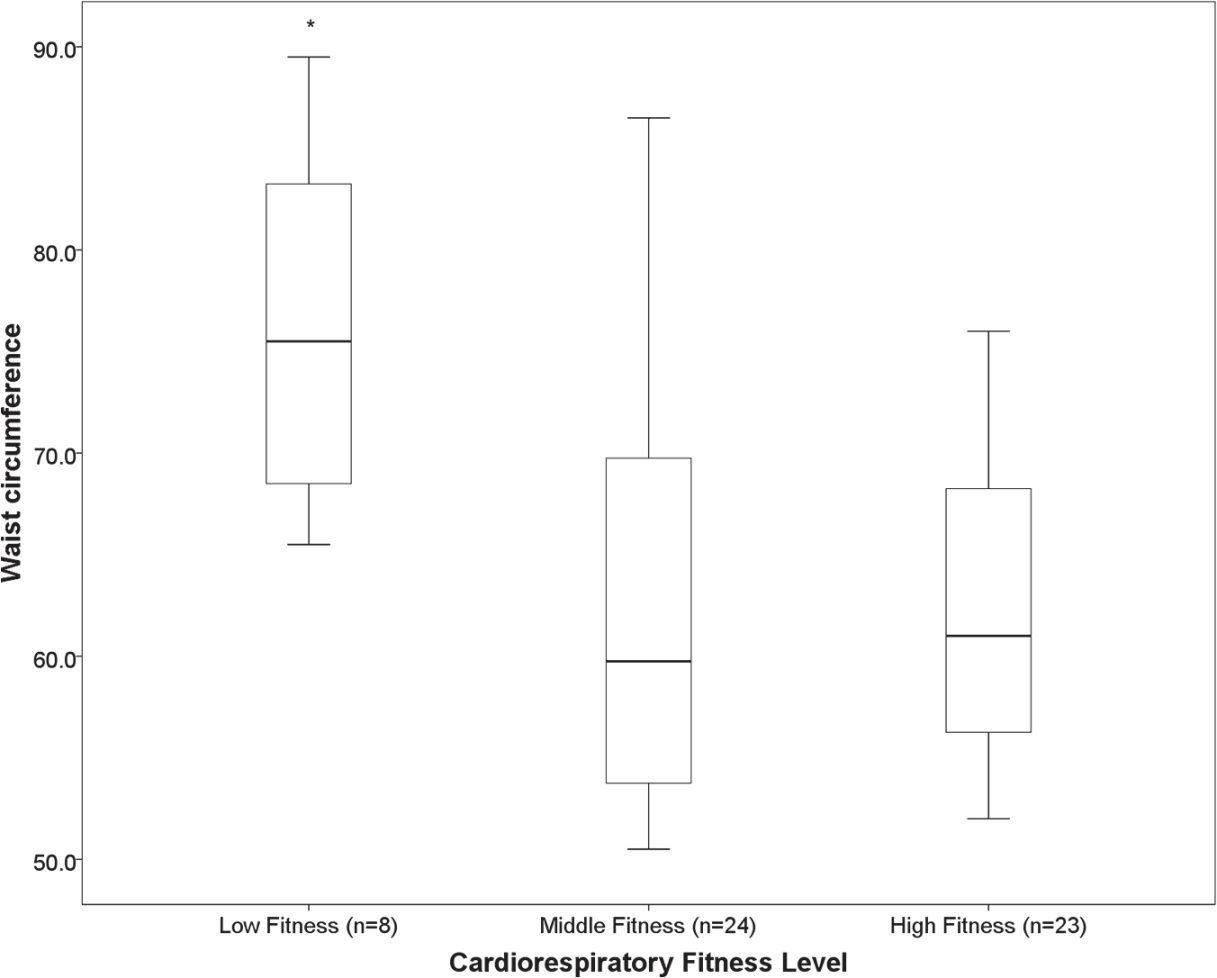
Mean waist circumference (cm) across tertiles of cardiorespiratory fitness. *Significantly different to “Middle” and “High” fitness groups, p<0.01.

**Fig 4:**
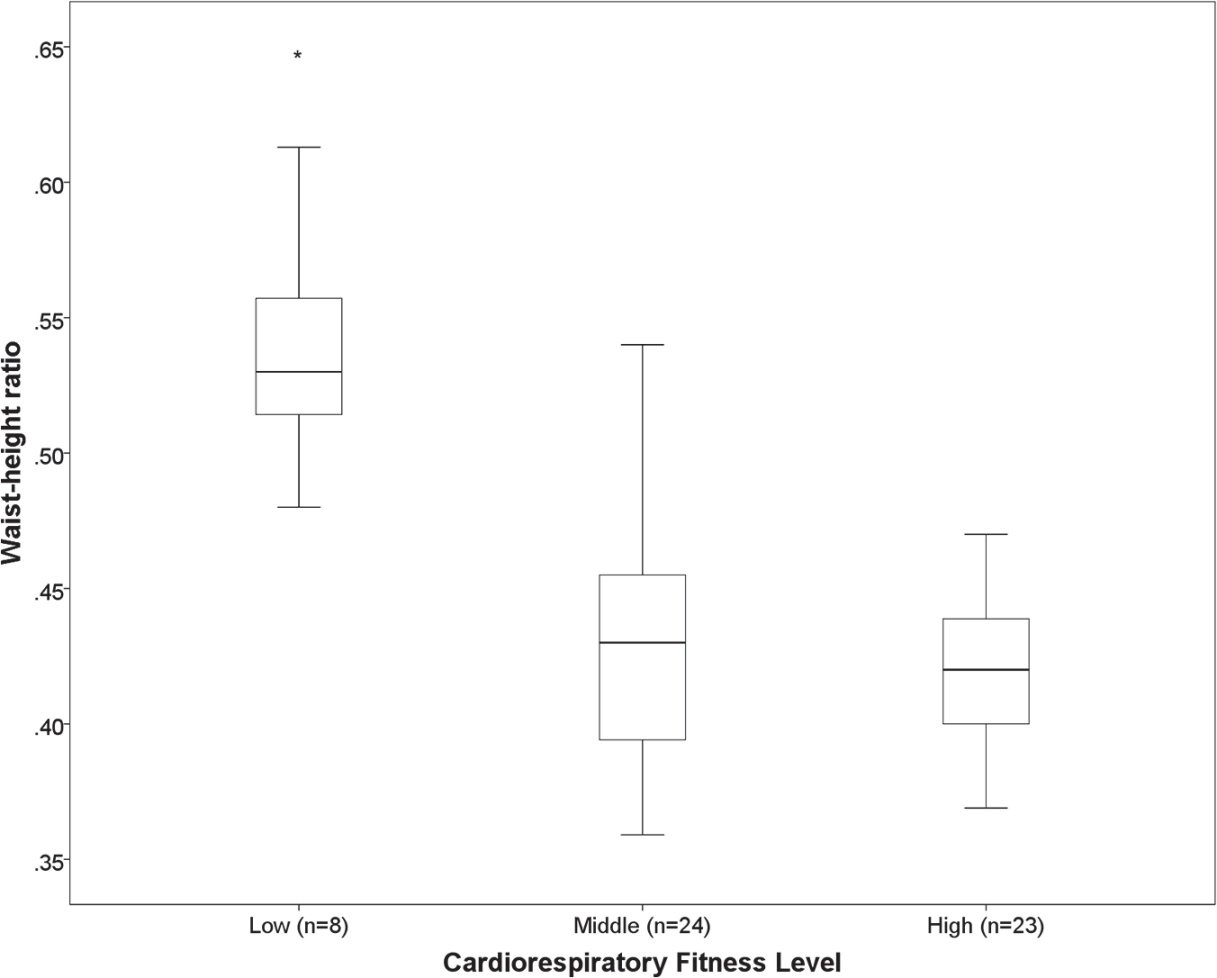
Mean waist-height ratio across tertiles of cardiorespiratory fitness. *Significantly different to “Middle” and “High” fitness groups, p<0.001.

There was no difference in percentage sedentary time, LPA, MPA, MVPA, mean counts.min^-1^, zSBP or zDBP across tertiles of cardiorespiratory fitness. Children in the most fit tertile spent significantly more time in VPA compared to children in the middle tertile (p <0.05) (Fig 1). Children in the least fit tertile had a significantly greater BMI grade, WC and WHtR than children in the middle and high fitness tertiles (Fig 2, Fig 3, Fig 4, respectively). Children in the middle fitness tertile had a greater BMI grade than children in the high fitness tertile (p<0.05) (Fig 2).

## Discussion

The results of this study suggest that vigorous activity but not light or moderate activity, is associated with cardiorespiratory fitness in children with CP. This finding is in agreement with research in children with typical development [18,19] and has implications for physical activity promotion among children with CP. Cardiorespiratory fitness was negatively associated with central adiposity, BMI and SBP. In addition, when the fitness of children was classified relative to the general pediatric CP population, children in the lowest fitness tertile had a significantly higher WC, WHtR and BMI than children in the middle and high tertile.

Children with CP are known to have low levels of cardiorespiratory fitness in comparison to their peers with typical development [1,3]. The findings of this study support the hypothesis that this may have a negative effect on their cardiometabolic health [5]. The associations between cardiorespiratory fitness and both WC and WHtR was stronger than that between cardiorespiratory fitness and BMI. These associations remained when the analyses were adjusted for BMI. The relationship between anthropometric measures and cardiorespiratory fitness is also strongest for WC in children with typical development [10]. This is likely because WC is an indicator of visceral adipose tissue [32]. Visceral adipose tissue is associated with a number of risk factors for cardiometabolic disease, including blood pressure, triglycerides, insulin resistance, and the metabolic syndrome in children with typical development [33]. Central adiposity has also been shown to have a stronger association with cardiometabolic risk factors, compared to BMI, in adults with CP [34–36]. Although cardiorespiratory fitness was associated with both central adiposity and SBP, the relationship between cardiorespiratory fitness and blood pressure was not independent of anthropometric measures. This suggests that a large proportion of the relationship between cardiorespiratory fitness and SBP in children with CP may be explained by the negative association between cardiorespiratory fitness and body fat.

Blood pressure and obesity are known to track from childhood to adulthood [37]. Children who don’t retain childhood risk factors into adulthood however, can reduce the likelihood of developing adult type II diabetes mellitus in young adulthood [38]. Implementation of risk factor screening and preventive programmes in childhood is therefore necessary to reduce the risk of adult cardiometabolic disease. Early intervention to prevent cardiometabolic disease may be even more important in children with CP who are known to have low levels of cardiorespiratory fitness in childhood [1], and participate in reduced levels of physical activity in adulthood [15].

The results of this study suggest that VPA may be required to improve cardiorespiratory fitness in children with CP. Although previous studies did not find a relationship between total physical activity and cardiorespiratory fitness in adults or children with CP [14–16] this study examined the associations of physical activity intensity with cardiorespiratory fitness. The cross-sectional design of this study however does not allow the direction of causality to be inferred. It is possible that children who are fit are more likely to participate in VPA. Although children are encouraged to reduce times spent in sedentary activity because of its association with cardiometabolic risk factors [21], sedentary activity was not associated with cardiorespiratory fitness in children with CP as we had hypothesised. This is in agreement with a recent study of children with typical development [17].

The relationship between VPA and cardiorespiratory fitness, independent of total activity, observed in this study has implications for the prescription of physical activity in children with CP. The current guidelines recommend that children with a disability accumulate 60 min of moderate-to-vigorous activity daily, where possible [39].There is no specific guideline for the accumulation of VPA. Children in the lowest fitness tertile in the current study accumulated on average 88.3 minutes of moderate activity per day, which was not significantly different to that accumulated by children with the highest fitness. This indicates that children with CP may achieve the physical activity guideline as a result of accumulating MPA, yet participate in low levels of VPA, potentially resulting in low cardiorespiratory fitness. Further research is required to optimise physical activity recommendations for children with CP.

The majority of VPA that children with typical development accumulate throughout the day is accumulated through sport. It is possible that reduced participation in sport among children with CP, even among those with minimal impairments [40,41], contributes to low levels of VPA. Personal and environmental factors that act as barriers to participation in sport among children and adolescents with CP include fear of exclusion, fear of losing, the perception that sport isn’t fun and a lack of teams that cater for children with a disability [42]. In order to improve and maintain cardiorespiratory fitness in children with CP a holistic rehabilitative approach is required to overcome these barriers to participation in sport and potentially increase habitual VPA.

There are limitations to this study including the cross-sectional design of the study, which as discussed previously does not allow the direction of causation to be established. Confounding factors such as diet, which was not assessed, may also have affected this association. Another limitation of this study is that oxygen uptake cannot be predicted from the level achieved on the shuttle run test. Levels of cardiorespiratory fitness presented in the current study are therefore relative to children with CP only and do not give an indication of the absolute fitness of the cohort. Further, due to the lack of validated maximal exercise tests for children in GMFCS levels III, IV and V at the time this study was conducted, the sample was limited to children in GMFCS levels I and II. The results of this study therefore cannot be generalised to the greater pediatric CP population.

## Conclusions

The results of this study demonstrate that children with CP who have low levels of cardiorespiratory fitness have high BMI, central adiposity, and elevated blood pressure. Vigorous physical activity, but not moderate or light activity, was positively associated with cardiorespiratory fitness. This indicates that healthcare professionals should promote participation in vigorous activity in order to improve cardiorespiratory fitness and the associated cardiometabolic risk profile of this population.

## References

[1] Verschuren O, Takken T. Aerobic capacity in children and adolescents with cerebral palsy. Res Dev Disabil. 2010;31:1352–1357. 10.1016/j.ridd.2010.07.005 20674266

[2] Capio CM, Sit CH, Abernethy B, Masters RS. Fundamental movement skills and physical activity among children with and without cerebral palsy. Res Dev Disabil. 2012;33:1235–1241. 10.1016/j.ridd.2012.02.020 22502850

[3] Unnithan VB, Clifford C, Bar-Or O. Evaluation by exercise testing of the child with cerebral palsy. Sports Med. 1998;26:239–251. 9820923 10.2165/00007256-199826040-00003

[4] Stevens SL, Holbrook EA, Fuller DK, Morgan DW. Influence of age on step activity patterns in children with cerebral palsy and typically developing children. Arch Phys Med Rehabil. 2010;91:1891–1896. 10.1016/j.apmr.2010.08.015 21112431 PMC3052963

[5] Fowler EG, Kolobe TH, Damiano DL, Thorpe DE, Morgan DW, Brunstrom JE, et al Promotion of physical fitness and prevention of secondary conditions for children with cerebral palsy: Section on Pediatrics Research Summit Proceedings. Phys Ther. 2007;87:1495–1510. 17895351 10.2522/ptj.20060116

[6] Dallmeijer AJ, Brehm MA. Physical strain of comfortable walking in children with mild cerebral palsy. Disabil Rehabil. 2011;33:1351–1357. 10.3109/09638288.2010.531374 21073360

[7] Opheim A, Jahnsen R, Olsson E, Stanghelle JK. Walking function, pain, and fatigue in adults with cerebral palsy: A 7-year follow-up study. Dev Med Child Neurol. 2009;51:381–388. 10.1111/j.1469-8749.2008.03250.x 19207296

[8] Janssen I, Cramp WC. Cardiorespiratory fitness is strongly related to the metabolic syndrome in adolescents. Diabetes Care. 2007;30:2143–2144. 17536078 10.2337/dc07-0734

[9] Brage S, Wedderkopp N, Ekelund U, Franks PW, Wareham NJ, Andersen LB, et al Features of the metabolic syndrome are associated with objectively measured physical activity and fitness in danish children: The European Youth Heart Study (EYHS). Diabetes Care. 2004;27:2141–2148. 15333475 10.2337/diacare.27.9.2141

[10] Klasson-Heggebo L, Andersen LB, Wennlof AH, Sardinha LB, Harro M, Froberg K, et al Graded associations between cardiorespiratory fitness, fatness, and blood pressure in children and adolescents. Br J Sports Med. 2006;40:25–9 16371486 10.1136/bjsm.2004.016113 PMC2491946

[11] Van den Berg-Emons RJ, Van Baak MA, Speth L, Saris WH. Physical training of school children with spastic cerebral palsy: Effects on daily activity, fat mass and fitness. Int J Rehabil Res. 1998;21:179–194. 9924680 10.1097/00004356-199806000-00006

[12] Verschuren O, Ketelaar M, Gorter JW, Helders PJ, Uiterwaal CS, Takken T. Exercise training program in children and adolescents with cerebral palsy: A randomized controlled trial. Arch Pediatr Adolesc Med. 2007;161:1075–1081. 17984410 10.1001/archpedi.161.11.1075

[13] Kulinski JP, Khera A, Ayers CR, Das SR, deLemos JA, Blair SN, et al Association between cardiorespiratory fitness and accelerometer-derived physical activity and sedentary time in the general population. Mayo Clin Proc. 2014;89:1063–71. 10.1016/j.mayocp.2014.04.019 25012770 PMC5152946

[14] Maltais DB, Pierrynowski MR, Galea VA, Bar-Or O. Physical activity level is associated with the O2 cost of walking in cerebral palsy. Med Sci Sports Exerc. 2005;37:347–353. 15741829 10.1249/01.mss.0000155437.45937.82

[15] Nieuwenhuijsen C, van der Slot WM, Dallmeijer AJ, Janssens PJ, Stam HJ, Roebroeck ME, et al Physical fitness, everyday physical activity, and fatigue in ambulatory adults with bilateral spastic cerebral palsy. Scand J Med Sci Sports. 2011;21:535–542. 10.1111/j.1600-0838.2009.01086.x 20459469

[16] Slaman J, Bussmann J, van der Slot WM, Stam HJ, Roebroeck ME, van den Berg-Emons RJ, et al Physical strain of walking relates to activity level in adults with cerebral palsy. Arch Phys Med Rehabil. 2013;94:896–901. 10.1016/j.apmr.2012.11.005 23149309

[17] Denton SJ, Trenell MI, Plotz T, Savory LA, Bailey DP, Kerr CJ. Cardiorespiratory fitness is associated with hard and light intensity physical activity but not time spent sedentary in 10–14 year old schoolchildren: The HAPPY study. PLoS One. 2013;8:e61073 10.1371/journal.pone.0061073 23577192 PMC3618292

[18] Dencker M, Thorsson O, Karlsson MK, Linden C, Wollmer P, Andersen LB. Daily physical activity related to aerobic fitness and body fat in an urban sample of children. Scand J Med Sci Sports. 2008;18:728–735. 10.1111/j.1600-0838.2007.00741.x 18248550

[19] Hay J, Maximova K, Durksen A, Carson V, Rinaldi RL, Torrance B, et al Physical activity intensity and cardiometabolic risk in youth. Arch Pediatr Adolesc Med. 2012;166:1022–1029. 10.1001/archpediatrics.2012.1028 22965682

[20] Palisano RJ, Rosenbaum P, Bartlett D, Livingston MH. Content validity of the expanded and revised gross motor function classification system. Dev Med Child Neurol. 2008;50:744–750. 10.1111/j.1469-8749.2008.03089.x 18834387

[21] Ryan JM, Hensey O, McLoughlin B, Lyons A, Gormley J. Reduced moderate-to-vigorous physical activity and increased sedentary behavior is associated with elevated blood pressure values in children with cerebral palsy. Phys Ther. 2014;94(8):1144–53. 10.2522/ptj.20130499 24700137

[22] [no authors] Obesity: Preventing and managing the global epidemic. report of a WHO consultation. World Health Organ Tech Rep Ser. 2000;894:i–xii, 1–253 11234459

[23] Cole TJ, Bellizzi MC, Flegal KM, Dietz WH. Establishing a standard definition for child overweight and obesity worldwide: International survey. BMJ. 2000;320:1240–1243. 10797032 10.1136/bmj.320.7244.1240 PMC27365

[24] Pan H, Cole TJ. LMSGrowth program version 2.77, a Microsoft Excel add-in to access growth references based on the LMS method. 2007.

[25] Stergiou GS, Yiannes NG, Rarra VC. Validation of the Omron 705 IT oscillometric device for home blood pressure measurement in children and adolescents: The Arsakion School Study. Blood Press Monit. 2006;11:229–234. 16810034 10.1097/01.mbp.0000209074.38331.16

[26] Jackson LV, Thalange NK, Cole TJ. Blood pressure centiles for Great Britain. Arch Dis Child. 2007;92:298–303. 16905566 10.1136/adc.2005.081216 PMC2083671

[27] Verschuren O, Takken T, Ketelaar M, Gorter JW, Helders PJ. Reliability and validity of data for 2 newly developed shuttle run tests in children with cerebral palsy. Phys Ther. 2006;86:1107–1117. 16879044

[28] Verschuren O, Bloemen M, Kruitwagen C, Takken T. Reference values for aerobic fitness in children, adolescents, and young adults who have cerebral palsy and are ambulatory. Phys Ther. 2010;90:1148–1156. 10.2522/ptj.20090318 20558568

[29] Vanhelst J, Beghin L, Rasoamanana P, Theunynck D, Meskini T, Iliescu C, et al Calibration of the RT3 accelerometer for various patterns of physical activity in children and adolescents. J Sports Sci. 2010;28:381–387. 20175015 10.1080/02640410903508821

[30] Ryan J, Walsh M, Gormley J. Ability of RT3 accelerometer cut points to detect physical activity intensity in ambulatory children with cerebral palsy. Adapt Phys Activ Q. 2014;31:310–324. 10.1123/apaq.2013-0088 25211479

[31] Myers RH. Classical and modern regression with applications Boston: Duxbury/Thompson Learning; 1990.

[32] Taylor RW, Jones IE, Williams SM, Goulding A. Evaluation of waist circumference, waist-to-hip ratio, and the conicity index as screening tools for high trunk fat mass, as measured by dual-energy X-ray absorptiometry, in children aged 3–19 y. Am J Clin Nutr. 2000;72:490–495. 10919946 10.1093/ajcn/72.2.490

[33] Kwon JH, Jang HY, Oh MJ, Rho JS, Jung JH, Yum KS, et al Association of visceral fat and risk factors for metabolic syndrome in children and adolescents. Yonsei Med J. 2011;52:39–44. 10.3349/ymj.2011.52.1.39 21155033 PMC3017706

[34] Ryan JM, Crowley VE, Hensey O, McGahey A, Gormley J. Waist circumference provides an indication of numerous cardiometabolic risk factors in adults with cerebral palsy. Arch Phys Med Rehabil. 2014;95(8):1540–1546. 10.1016/j.apmr.2014.03.029 24742941

[35] Peterson MD, Haapala HJ, Chaddha A, Hurvitz EA. Abdominal obesity is an independent predictor of serum 25-hydroxyvitamin D deficiency in adults with cerebral palsy. Nutr Metab (Lond). 2014;11:22-7075-11-22. eCollection 2014. 10.1186/1743-7075-11-22 PMC4039320 24883075

[36] Peterson MD, Haapala HJ, Hurvitz EA. Predictors of cardiometabolic risk among adults with cerebral palsy. Arch Phys Med Rehabil. 2012;93:816–821. 10.1016/j.apmr.2011.12.024 22541309

[37] Juhola J, Magnussen CG, Viikari JS, Kahonen M, Hutri-Kahonen N, Jula A, et al Tracking of serum lipid levels, blood pressure, and body mass index from childhood to adulthood: The Cardiovascular Risk in Young Finns study. J Pediatr. 2011;159:584–590. 10.1016/j.jpeds.2011.03.021 21514597

[38] Morrison JA, Glueck CJ, Woo JG, Wang P. Risk factors for cardiovascular disease and type 2 diabetes retained from childhood to adulthood predict adult outcomes: The Princeton LRC follow-up study. Int J Pediatr Endocrinol. 2012;2012:6-9856-2012-6. 10.1186/1687-9856-2012-6 PMC3466140 22507454

[39] WHO. Global recommendations on physical activity for health. 2010. 26180873

[40] Imms C, Reilly S, Carlin J, Dodd K. Diversity of participation in children with cerebral palsy. Dev Med Child Neurol. 2008;50:363–369. 10.1111/j.1469-8749.2008.02051.x 18355337

[41] Mc Manus V, Corcoran P, Perry IJ. Participation in everyday activities and quality of life in pre-teenage children living with cerebral palsy in south west Ireland. BMC Pediatr. 2008;8:50-2431-8-50. 10.1186/1471-2431-8-50 PMC2585080 18976459

[42] Verschuren O, Wiart L, Hermans D, Ketelaar M. Identification of facilitators and barriers to physical activity in children and adolescents with cerebral palsy. J Pediatr. 2012;161:488–494. 10.1016/j.jpeds.2012.02.042 22494875

